# Sustainable psychiatry in the UK

**DOI:** 10.1192/pb.bp.113.045054

**Published:** 2014-12

**Authors:** Sucharita Yarlagadda, Daniel Maughan, Susie Lingwood, Phil Davison

**Affiliations:** 1 Oxford Deanery, Oxford; 2 Royal College of Psychiatrists, London; 3 Oxford Health NHS Foundation Trust, Oxford; 4 Barnet, Enfield and Haringey Mental Health Trust, London

## Abstract

Demands on our mental health services are growing as financial pressures increase. In addition, there are regular changes to service design and commissioning. The current political mantra is ‘more and more, of better quality, for less and less, please’. We suggest that mental health services need to actively respond to these constraints and that clinical transformation is needed to move towards a more sustainable system of healthcare. Emphasis on prevention, patient empowerment and leaner, greener services is required alongside more extensive use of technologies. Focusing on these areas will make mental health services more responsive to the challenges we face and serve to future-proof psychiatry in the UK. Services need to be delivered to provide maximum benefit to the health of our patients, but also to our society and the environment.

Sustainable healthcare, as a concept, describes a healthcare system ‘that is environmentally, economically and socially viable indefinitely, that works harmoniously both with the human body and the non-human environment, and which does not cause any significant unfair or disproportionate effects which may hinder the functioning, development or viability of the healthcare system itself.’^1^ In other words, sustainability in healthcare refers to the balance between the economic, environmental and social constraints and demands within healthcare settings. Sustainable development refers to the efforts made by all those within healthcare to maintain this balance while modernising and improving healthcare systems and modes of delivery. The concept of sustainability in healthcare has developed in response to the need to find a viable alternative to the current medical paradigm of a pharmaceutically dominant mental healthcare system that pays inadequate attention to prevention. This paradigm has led to rising economic, social and environmental costs,^2^ and has resulted in a healthcare system without enough focus on creating healthy lifestyles and communities.

A sustainable healthcare system that recognises and addresses the broader environmental and societal impact of mental illness is crucial to the long-term sustainability of high-quality healthcare provision, in an era of ever-diminishing resources. Four principles of sustainable healthcare have been identified:^3^ prevention; patient empowerment; lean service delivery; and preferential use of low-carbon technologies (such as telecare or remote monitoring of patient symptoms using smartphone apps). Widespread implementation of these principles will ensure mental health services maintain high-quality standards despite ongoing constraints.

Despite dramatic improvements over the past 50 years in diagnosing mental illness, with improved availability of new treatments and better outcomes, mental health services still face some key challenges. A recent inquiry identified six key themes that mental health services will need to address to become fit for purpose in the 21st century.^4^

1 Personalising services through the engagement of patients, their carers and families, with an emphasis on self-management.

2 Integrated care between physical health, mental health and social care.

3 Addressing an individual’s mental health needs across the lifespan.

4 Integrating mental health training into the general workplace and in other professions, such as teaching, alongside encouraging peer support workers.

5 Research into both clinical and social interventions to support people with mental health problems.

6 Public mental health with a focus on prevention.

Addressing these issues with limited resources is challenging. In this article we will describe how taking a sustainable approach by understanding the environmental, economic and social impacts of mental health services, called the ‘triple bottom line’ approach, is an opportunity to rethink mental health service provision in a way that can address these constraints. This article is not a comprehensive review of all the interventions available that will ensure sustainable mental health services, but provides an overview, with the hope of encouraging sustainable thinking among mental health professionals.

## Economic cost of mental health services

Mental health problems are the largest single cause of illness in the UK, accounting for 23% of the total burden of disease, and in people of working age are the health conditions with the most impact.^5^ Mental illness has been found to be more debilitating than most physical illnesses, for example, depression causes more disability than angina, arthritis, asthma or diabetes.^6^ Nevertheless, only 11.1% of the National Health Service (NHS) budget (£11.9 billion) is spent on treating mental health problems,^7^ while receiving only 5% of the total for health research.^3^ This is worsened by the rising costs of mental health services – antidepressants have seen the largest increase in both net ingredient cost (£49.8 million or 22.6%) and the number of items dispensed (£3.9 million or 9.1%) of any section in the *British National Formulary* between 2010 and 2011.^8^

The economic and social costs of mental ill health in England are estimated at around £105 billion per annum, including £21.3 billion in health and social care costs. It is estimated that these costs could double over the next 20 years.^2^ Prevention is key to addressing these spiralling costs.

A leaner mental health service is also needed to ensure high-quality care with lower costs. A lean mental health service will provide the right things to the right place at the right time in the right quantities, by evidence-based decision-making, while minimising waste, reducing delays and being flexible and open to change.^9^ Simplified care delivery with reduced use of low-value activities (poor health outcomes with high costs) will result in significant savings. Design of care pathways should include high-value activities^10^ that take advantage of synergies in treating comorbid physical and mental health conditions together.

## Environmental cost of mental health services

Environmental costs are included in sustainable (triple bottom line) approaches because environmental change has been globally recognised as the greatest single threat to human health in the 21st century^11^ and is principally caused by human activity. The UK government has responded by introducing regulations for large organisations, including the NHS, with the aim of reducing environmental impacts.

The carbon footprint for NHS England in 2007 was 21 million tonnes of CO_2_ equivalent (CO_2_e).^12^ Mental health services account for 1.47 million tonnes of CO_2_e^13^ and are out-performing other services when it comes to carbon footprint per pound spent or per disability-adjusted life-year (DALY) saved.^14^ This may be due to the nature of mental health service provision relying less on resource-heavy interventions such as dialysis in kidney care, with more emphasis on service-based interventions such as psychotherapy.

The NHS has demonstrated good leadership in reducing the carbon footprint through the Good Corporate Citizenship model and the sustainable development strategy.^15,16^ These aim to meet government targets of a 10% reduction of the 2007 NHS carbon footprint by 2015 and the Climate Change Act 2008 target of a 34% reduction in emissions by 2020. The government has also backed the Carbon Reduction Commitment Scheme, an emissions trading scheme for the UK that covers public sector organisations, making it a legal requirement for large trusts to reduce their emissions or face penalties.^17^ The challenge has therefore been set and the onus is on the NHS to respond.

Meeting these carbon reduction targets will require a transformation in the way mental health services are designed, delivered and evaluated, as the main source of the carbon footprint of a health service is not its buildings or energy use but factors relating to clinical practice.^18^ Although there is good reason for mental health services to reduce the carbon footprint of their estates as this releases cash rapidly, energy usage only accounts for 22% of the carbon footprint of the NHS.^18^ Procurement (the purchase of medication and medical equipment) accounts for the majority of the carbon footprint (60%) of the NHS,^18^ so NHS trusts need to ensure that medications and equipment are sourced sustainably. Crucially though, clinicians need to improve the sustainability of their individual practice. This requires a re-assessment of current services and interventions using the four principles of sustainable healthcare noted earlier.

## Social cost of mental health services

Poor mental health has a large impact on the lives of individuals, their families, friends, carers and communities. Most mental health problems start early in life, with half of those affected experiencing symptoms by the age of 14,^19^ and three-quarters by their mid-20s.^20^ Economic evaluations of mental illness during childhood and adolescence show costs to society ranging from £11 030 to £59 130 annually per child.^21^

People with mental illnesses often have lower educational attainment,^22^ find it harder to obtain and stay in work,^23^ are more likely to be homeless,^24^ have lower incomes,^25^ and are more likely to live in areas of high social deprivation.^26^ They are more likely to have poor physical health, with high rates of smoking, alcohol and substance misuse^27^ and, on average, die 25 years earlier than the general population if they have a serious mental illness.^28^

Social costs include costs to Social Services, costs of informal care, costs of unemployment, sickness absence costs, costs to criminal justice and probation services. Sickness absence due to mental ill health costs £8.4 billion per year. Replacing staff who leave their posts because of mental illness costs employers £2.4 billion a year^29^ and 43% of those on long-term benefits due to health issues have a primary mental health problem.^30^

Given these large costs, it is essential that we move towards incorporating measures of social sustainability. When designing, delivering and evaluating interventions, factors such as employment and appropriate housing should be taken into account, alongside symptomatic changes. These factors could demonstrate benefit in terms that are more ‘health’ focused than ‘illness’ focused. Addressing the social impact of mental health services is critical as poor mental health has an impact on society and elements of society can contribute to poor mental health. Restoring social capital should be seen as a core responsibility of mental health services. Realising the full benefits of treating mental illness includes the restoration of a person’s social life and integration with their community.

## Benefits and synergies of sustainable psychiatry

The importance of interaction with the natural environment for achieving good mental health has been well recognised.^31^ Moving to a better quality physical environment can lead to improvements in psychological well-being, social relationships, performance in school,^32^ and a reduction in anxiety and depression.^33^ Greener lifestyles such as walking, cycling and access to green spaces are beneficial to physical health. They also improve mental health by reducing depression, reducing admissions and reducing symptoms in young people with attention-deficit hyperactivity disorder (ADHD).^34–37^ The UK Faculty of Public Health states that ‘Safe, green spaces may be as effective as prescription drugs in treating some forms of mental illness, without the ”costs” of side-effects and ever rising numbers of prescriptions’.^37^ Direct contact with nature leads to a greater sense of connectedness to the community.^38^ Spending time in natural areas has been associated with speedier recovery from illness,^39^ stress reduction, alleviation of anxiety symptoms and reduction of psychotic symptoms.^40–42^ Children exposed to green settings feel more relaxed, less stressed, more positive and able to cope, and have improved cognitive functioning and social connectedness.^43,44^ In adults, contact with nature has been shown to reduce aggressive behaviour and violence in cities.^45^ Later in life, exposure to the natural environment is associated with decreased agitation and aggression in patients with late-stage dementia.^46^ Views of natural environments improve post-operative recovery, with lower fear and anger levels in those experiencing stress.^41,47^ Physical activity can be an effective supplement to treatment in mild and moderate depression and anxiety disorders.^48,49^ There is, therefore, clearly a process whereby physical activity improves mental health, not only through improving physical health, but also directly through reducing psychiatric symptoms.^50^

In contrast, poor housing quality,^51^ living in poor-quality neighbourhoods (e.g. areas vulnerable to vandalism, break-ins, unsafe streets)^52^ and high-rise ‘inner city’ accommodation^53^ are all associated with poor mental health.

Cognitive–behavioural therapy (CBT) is a good example of a sustainable intervention (Box 1). It has good clinical evidence and is as effective as drugs in the short term for anxiety conditions and depression, but it is also more effective in preventing relapse.^44^ CBT is also cost-effective and potentially has a very low carbon footprint. An example of an innovative sustainable intervention is True Colours (www.truecolours.nhs.uk), an online self-management service for monitoring symptoms and mood (Box 2).

**Box 1** Example of an established sustainable intervention: CBT

Improving Access to Psychological Therapies (IAPT), which mostly uses the cognitive–behavioural therapy (CBT) approach, is estimated to have paid for itself through reduced physical healthcare costs, increased employment and subsequent reduced disability benefits and extra tax receipts.^44^ In the first 2 years after the end of CBT the benefits per person treated include the extra gross domestic product produced of around £1200, National Health Service (NHS) savings in physical and mental health service usage of £300, and reduced suffering equating to quality-adjusted life-years (QALYs) worth £3300. These gains far exceed the one-off cost of CBT (£750). The overall gain to the Exchequer in terms of employment, reduced benefits and increased taxes is around £900 plus the NHS savings of £300. Thus the cost is fully repaid.^54^ Evidence of the cost-effectiveness of psychological therapy is also available in medical conditions such as chronic obstructive pulmonary disease,^55,56^ angina,^57^ diabetes^58^ and arthritis,^59^ where costs for CBT are more than offset by the NHS savings on physical healthcare costs.

**Box 2** Example of a novel sustainable intervention: True Colours

True Colours allows people with mental health conditions to regularly monitor their symptoms by means of texting in their symptoms or inputting them straight on to the True Colours website (www.truecolours.nhs.uk). Symptoms are scored according to a series of self-rated measures for the different symptom domains, mood, anxiety, etc. A graphical representation of symptoms over time is then compiled, and is accessible online by the patient, with relevant life events included. Graphs can then be examined at a later date with a clinician, either face to face or on the telephone. This simple intervention has the potential to transform the way mental health services are run, reducing appointments, reducing admissions and empowering patients to self-manage. These potential changes could have a significant impact on the environmental footprint of the service by reducing service usage. Also, by enabling patients to take a more active role in the assessment and management of their mental health condition, the service creates a more patient-centred focus.

## Recommendations for improving sustainability

- Shift focus of mental health services towards prevention of mental illness among individuals and communities across the life course. Optimal treatment for mental disorders will only be able to avert 28% of the burden of mental illness; prevention is needed to tackle the other 72%.^60^ Focusing on prevention provides value for money. For example, prevention of conduct disorders could save £150 000 per case in lifetime costs, whereas promoting positive mental health in children with moderate mental health issues could yield benefits over the lifetime of £75 000 per case.^61^
- Priority setting should be based on an assessment of the disease burden and its contribution to health inequalities, using cost-effective, high-value, sustainable treatments for

**Fig 1:**
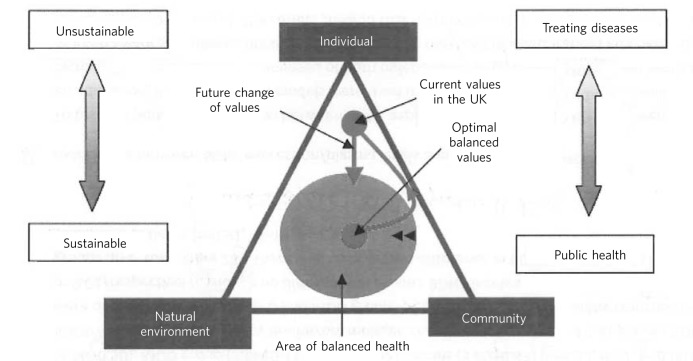
Area of balanced health. This diagram explains the values held by society. The triangle demonstrates the three main values of individual, community and environment (natural). The circle represents the area where the values are in a balanced proportion leading to the broader definition of health and well-being. As we have urbanised over the past 20 years, we have shifted our values away from community and environment towards the individual. Valuing the individual at the expense of the environment and community is not only less sustainable, but favours healthcare that treats disease rather than promoting supportive communities and environments. To regain a sense of well-being it is argued that we should change our values and reconnect with the natural environment and community in which we live and work.

which good evidence is available, while decommissioning those with low or uncertain value.
- Integrated care should be standard alongside collaborative working with patients, carers and families in co-developing treatment plans to encourage ownership of health.
- Early identification will enable significant savings for health services. Estimates show that for every one pound spent there are substantial savings to be made by health and social services: early detection and intervention for psychosis (£17.97 saved), early diagnosis and treatment of depression at work (£5.03 saved), suicide awareness training to general practitioners (GPs) (£43.99 saved), suicide prevention through bridge safety barriers (£54.45 saved).^62^
- Local government should prioritise health promotion as it shows a favourable effect on survival.^63^ Subjective well-being increases life expectancy by 7.5 years and provides a similar degree of protection from heart disease to giving up smoking. It also improves recovery and health outcomes in many chronic diseases.^63^
- Investing in research that will enable the design of evidence-based models of care that take into account environmental and social impacts of healthcare.

Figure 1 demonstrates how balanced mental health is sustainable and public-health focused.

## Conclusions

Developing a sustainable approach to our clinical practice in mental health is a crucial step in ensuring mental health services will continue to provide high-quality care in the 21st century. Service improvement activities that are evidence based and self-financing from a healthcare perspective are already available, but show even greater benefits when we include the wider society. Although certain interventions provide pay-offs in the short term, we need to understand that interventions such as those in childhood take many years to show effects. The key principles of clinical transformation – prevention, self-care, lean services and the use of low-carbon technologies – need to feature prominently in any future mental health strategy and policy. A small shift in spending from treatment to prevention and promotion is likely to enhance efficiency gains, but many of these innovations require working with other organisations outside the NHS. This approach requires a broadening of understanding of both the costs and the wider benefits of *all* interventions and services in mental health. Ideally, commissioners should move from using QALYs to a more sustainable cost analysis measure incorporating the relative contribution of social, economic and environmental determinants of mental health. Investment should be directed towards developing this approach. This will lead to greater awareness among clinicians about the full impact of each clinical decision they make and each intervention they implement.

Sustainable mental health is not a utopian vision. It is directly linked to quality of care, patient safety, patient and staff experience, population well-being, risk management, organisational reputation, community benefit and cost savings. For those who want to know more or get involved, please join Psych Susnet, the sustainability network for mental health professionals at http://sustainablehealthcare.org.uk/mental-health-susnet
